# V1V2-specific complement activating serum IgG as a correlate of reduced HIV-1 infection risk in RV144

**DOI:** 10.1371/journal.pone.0180720

**Published:** 2017-07-05

**Authors:** Lautaro G. Perez, David R. Martinez, Allan C. deCamp, Abraham Pinter, Phillip W. Berman, Donald Francis, Faruk Sinangil, Carter Lee, Kelli Greene, Hongmei Gao, Sorachai Nitayaphan, Supachai Rerks-Ngarm, Jaranit Kaewkungwal, Punnee Pitisuttithum, James Tartaglia, Robert J. O’Connell, Merlin L. Robb, Nelson L. Michael, Jerome H. Kim, Peter Gilbert, David C. Montefiori

**Affiliations:** 1 Duke University Medical Center, Durham, North Carolina, United States of America; 2 Fred Hutchinson Cancer Research Center, Seattle, Washington, United States of America; 3 Public Health Research Institute, Newark, New Jersey, United States of America; 4 Baskin School of Engineering, University of California, Santa Cruz, California, United States of America; 5 Global Solutions for Infectious Diseases, South San Francisco, California, United States of America; 6 Armed Forces Research Institute of Medical Sciences, Bangkok, Thailand; 7 Ministry of Public Health, Bangkok, Thailand; 8 Faculty of Tropical Medicine, Mahidol, Thailand; 9 Department of Research and Development, Sanofi Pasteur, Swiftwater, Pennsylvania, United States of America; 10 Military HIV Research Program, Walter Reed Army Institute of Research, Silver Spring, Maryland, United States of America; 11 International Vaccine Institute, Seoul, Republic of Korea; Emory University School of Medicine, UNITED STATES

## Abstract

Non-neutralizing IgG to the V1V2 loop of HIV-1 gp120 correlates with a decreased risk of HIV-1 infection but the mechanism of protection remains unknown. This V1V2 IgG correlate was identified in RV144 Thai trial vaccine recipients, who were primed with a canarypox vector expressing membrane-bound gp120 (vCP1521) and boosted with vCP1521 plus a mixture gp120 proteins from clade B and clade CRF01_AE (B/E gp120). We sought to determine whether the mechanism of vaccine protection might involve antibody-dependent complement activation. Complement activation was measured as a function of complement component C3d deposition on V1V2-coated beads in the presence of RV144 sera. Variable levels of complement activation were detected two weeks post final boosting in RV144, which is when the V1V2 IgG correlate was identified. The magnitude of complement activation correlated with V1V2-specific serum IgG and was stronger and more common in RV144 than in HIV-1 infected individuals and two related HIV-1 vaccine trials, VAX003 and VAX004, where no protection was seen. After adjusting for gp120 IgA, V1V2 IgG, gender, and risk score, complement activation by case-control plasmas from RV144 correlated inversely with a reduced risk of HIV-1 infection, with odds ratio for positive versus negative response to TH023-V1V2 0.42 (95% CI 0.18 to 0.99, p = 0.048) and to A244-V1V2 0.49 (95% CI 0.21 to 1.10, p = 0.085). These results suggest that complement activity may have contributed in part to modest protection against the acquisition of HIV-1 infection seen in the RV144 trial.

## Introduction

The ALVAC-HIV (vCP1521) prime and recombinant gp120 AIDSVAX B/E + vCP1521 boost vaccine reduced the risk of HIV-1 infection by an estimated 31.2% compared to placebo in the RV144 efficacy trial in a community-based population in Thailand [1]. Reduced infection risk was significantly associated with total plasma IgG binding to a murine leukemia virus gp70 scaffold containing HIV-1 gp120 variable regions 1 and 2 (gp70-V1V2) [2, 3]. A similar correlation was seen with total plasma IgG binding to linear V2 peptides [4], and with plasma IgG3 binding to gp70-V1V2 scaffolds [5]. These V1V2 antibodies appear to bind the mid-loop region of V2 with a strong dependency on lysine (K) at position 169 and valine (V) at position 172 [6, 7]. Consistent with these findings, two genetic sieve analyses of RV144 breakthrough viruses found increased efficacy against viruses containing lysine (K) at position 169 [8, 9]. Because virus-specific CD8^+^ T cells [2] and tier 2 virus neutralizing antibodies [10] were nearly absent in this trial, the hypothesis has been raised that protection was mediated by non-neutralizing antibodies [11, 12]. In this regard, results of several RV144 follow-up studies implicate a role for non-neutralizing, Fc receptor (FcR)-mediated antibody effector functions [13, 14], including antibody-dependent cellular cytotoxicity (ADCC) [15–18] and phagocytosis [13].

The Fc region of IgG also has potential to activate the complement system of soluble proteins and cellular receptors that link innate and acquired immunity, which constitute a first line of defense against invading pathogens ([19], review). Complement activation can occur through three distinct pathways: classical, alternative and lectin, all of which converge at the activation of C3 convertase to generate C3 cleavage fragments. C3 cleavage precedes the formation of C5 convertases and assembly of the membrane attack complex (MAC), which forms lytic pores in the membranes of pathogens and infected cells. Antibody-mediated complement activation by HIV-1 is well-documented and can occur through both the classical and alternative pathways [20–22]. It has been suggested that HIV-1 is susceptible to complement-mediated lysis and inactivation [23–26] but other studies showing that HIV-1 infection is enhanced by complement in cells that co-express CD4 and complement receptors [27–35] indicate that lysis is limited and does not have a major impact on infectious virions. Resistance to complement lysis has been linked to one or more host cell-derived complement regulatory proteins (e.g., CD55, CD56, CD59) that are retained by HIV-1 during budding to prevent terminal complement pathway activation and MAC formation [36, 37]. Additional soluble factors, such as complement factor H, may further contribute to the ability of HIV-1 to evade complement lysis [38, 39]. In the absence of lysis, complement-opsonized HIV-1 is free to bind a variety of complement receptor-bearing cells, most notably cells expressing either CR1/CD35 [40, 41] or CR2/CD21 [42–44]. Cellular interactions of complement-opsonized HIV-1 could have a number of consequences that may be either beneficial or harmful to the host [20–22].

Here a customized multiplex assay was used to examine complement activation by V1V2-specific IgG in plasma from HIV-1-infected individuals and from vaccine recipients in RV144 and two related HIV-1 vaccine efficacy trials, VAX003 [45] and VAX004 [46], in which no protection was seen. This effort included an assessment of case-control plasma samples from RV144 to determine whether V1V2-specific complement-activating IgG was a correlate of infection risk.

## Material and methods

### Ethics statement

This study utilized pre-existing, de-identified specimens and was conducted under the approval of the local Institutional Review Boards (IRBs). The IRBs that conducted oversight for the respective sites are as listed previously [4]. The data were analyzed anonymously.

### Serum and plasma samples

Serum and plasma samples were obtained from the RV144, VAX003 and VAX004 HIV-1 vaccine efficacy trials (registration numbers NCT00223080, NCT0006327 and NCT00002441, respectively, ClinicalTrials.gov). RV144 tested two inoculations (weeks 0, 4) with a recombinant canarypox vector (vCP1521) expressing Gag and Pro of HIV-1 MN (subtype B), and membrane-linked gp120 from strain 92TH023 (CRF01_AE), followed by two boosts at weeks 12 and 24 with vCP1521 plus bivalent gp120 protein (AIDSVAX B/E, clade B strain MN + CRF01_AE strain A244) [1]. Plasma samples were obtained pre-immunization (week 0, visit 1) and 2 weeks after the final inoculation (week 26, visit 8) from a subset of vaccine recipients for assay development. Case-control samples comprised visit 8 plasma from 41 vaccine recipients (cases) who acquired HIV-1 infection after week 26, and from an additional 205 vaccine recipients (controls) selected randomly among those who had not acquired infection by the end of the trial (month 42). Additional week 26 plasmas from 20 placebo recipients who acquired HIV-1 infection after week 26, and 20 placebo recipients who remained uninfected at the end of the trial were included as negative controls in the case-control analysis. VAX003 tested seven inoculations with gp120 protein alone (AIDSVAX B/E, months 0, 1, 6, 12, 18, 24, and 30) in a cohort of mostly injection drug using men in Thailand [45]. VAX004 tested seven inoculations with gp120 protein (AIDSVAX B/B, clade B strains MN and GNE8) at months 0, 1, 6, 12, 18, 24, and 30 in mostly men who have sex with men in North America and Europe [46]. Serum samples from VAX003 and VAX004 were obtained at baseline (visit 2) and month 12.5 (visit 9) from trial participants who were uninfected at month 12. All clinical trials were conducted in accordance with the Declaration of Helsinki and local institutional review board requirements. Written informed consent was obtained from all clinical trial subjects. HIV-1-positive plasmas from antiretroviral drug-naïve individuals were obtained from Thailand's National Blood Bank and were confirmed by Env sequence analysis to be from subjects infected with CRF01_AE HIV-1 [47]. Normal human serum (NHS) used as a source of complement was purchased from Sigma (Cat. No. S1764), as was C3-deficient human serum (Sigma Cat. No. C8788).

### Monoclonal antibodies and gp70-V1V2 scaffolds

V1V2-specific monoclonal antibodies CH58, CH59 and HG107 were isolated from RV144 vaccine recipients and produced as IgG1 as described elsewhere [15]. HG118 is another V1V2-specific monoclonal antibody isolated from an RV144 vaccine recipient and produced as IgG1 (unpublished). The V1V2 envelope sequences from HIV-1 isolates 92TH023, A244, MN and Ce1086 were expressed as fusion proteins with the first 263 amino acids of the murine leukemia virus gp70 glycoprotein (gp70) as previously described [48].

### Customized multiplex assay to measure complement activation

Carboxylated microspheres (5x10^6^, Luminex, Cat. No. MC10043) were coupled with 25 μg of gp70-V1V2 scaffolds by covalent N-hydroxysulfosuccimide-ester linkages using a combination of 1-Ethyl-3-(3-dimethylaminopropyl) carbodiimide HCl (EDC) and N-hydroxysulfosuccimide (ThermoScientific) in phosphate buffered saline (PBS), pH 7.4 according to the manufacturer’s instructions. C3d complement deposition on gp70-V1V2 coated beads was performed in 96 well black plates (Bio-Rad, Cat. No. 171025001). To obtain a final dilution of 1:30, samples and NHS were separately diluted 1:7.5 in PBS and 25 μl of each were added to duplicate wells containing 2,500 antigen-coated beads in 50 μl of PBS. After incubation at room temperature for 1 hour the beads were washed with Bioplex buffer (BioRad, Cat. No. 171304500) and incubated with 100 μl of a 1:500 dilution of a biotinylated mouse monoclonal antibody to human C3d (Quidel, Cat. No. A702) at room temperature for 30 minutes. The beads were washed with Bioplex buffer and incubated at room temperature with 100 μl of a 1:500 dilution of PE-Streptavidin (BD Pharmingen, Cat. No. 554061) for 30 minutes. After a final wash with Bioplex buffer, beads were resuspended in 100 μl of Bioplex buffer and mean fluorescence intensity (MFI) was determined using MAGPIX fluorescence imagery (Luminex).

### Customized multiplex assay to measure V1V2 binding antibodies

IgG binding to gp70-V1V2-coated beads was measured in 96 well black plates (Bio-Rad, Cat. No. 171025001). Antigen-coated beads were suspended in PBS at a density of 50,000 beads/ml and 50 μl (2,500 beads) was mixed with an equal volume of a 1:50 dilution of the serum samples (total volume 100 μl/well) to obtain a 1:100 final dilution of the samples. After 1 hour incubation at room temperature, beads were washed with Bioplex buffer and incubated with 100 μl of a 1:2,500 dilution of biotin-conjugated rabbit anti-human IgG (heavy and light chain specific, Thermo Scientific, Cat. No. OK1781367) for 30 minutes at room temperature. The beads were washed with Bioplex buffer and incubated at room temperature with 100 μl of a 1:500 dilution of PE-Streptavidin (BD Pharmingen, Cat. No. 554061) for 30 minutes. After a final wash with Bioplex buffer, beads were resuspended in 100 μl of Bioplex buffer and the mean fluorescence intensity (MFI) was determined using the MAGPIX system (Luminex).

### Neutralizing antibody assay

Neutralizing antibodies were measured against Env-pseudotyped viruses TH023.6 and MN.3 in TZM-bl cells as described [49]. Neutralization titers were defined as the sample dilution at which relative luminescence units (RLU) were reduced by 50% compared to virus control wells after subtraction of background RLUs. Assay stocks of Env-pseudotyped viruses were prepared by co-transfection of an Env-expressing plasmid and an Env-defective backbone plasmid (pSG3Δenv) in 293T cells and titrated in TZM-bl cells [49].

### Statistical analysis

Complement activation readouts at week 26 were studied as correlates of risk of HIV-1 infection in vaccine recipients over the subsequent 3 years of follow-up using logistic regression models, as described in Haynes *et al*. [2]. All models adjusted for baseline behavioral risk score and gender. The models were studied with complement activation specified as either i) a quantitative variable (log-transformed and scaled to have SD = 1); ii) as a dichotomous variable of positive vs. negative response, with positive defined by a value greater than 3 standard deviations above the sample mean of the n = 20, week 26 uninfected placebo group readouts; or iii) a trichotomous variable defined as negative vs. medium vs. high response, where positive responses were dichotomized into either medium or high response based on thresholding the response at 1,000 MFI for TH023 or 100 for A244 and MN.3. Three types of models were fit, either adjusting for both the primary IgA and primary IgG V1V2 variables that were previously identified as independent correlates of risk [2], adjusting for only IgA, or adjusting for neither of these variables. Scatterplots of the complement activation variables versus one another and versus the primary IgA and primary IgG V1V2 variables are reported, together with Spearman rank correlations.

## Results

### Complement activation assay

**Fig 1:**
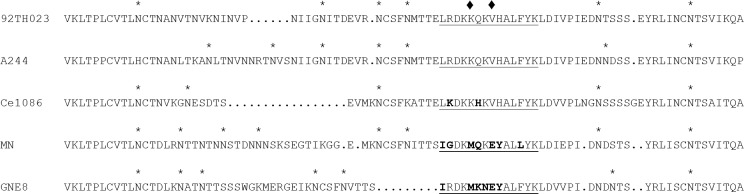
V1V2 sequences in the gp70 scaffolds. Shown is an amino acid sequence alignment of the V1V2 and flanking regions used as gp70-V1V2 scaffolds (with the exception of GNE8) to assess complement-activating antibodies. The hotspot peptide region (aa 165–178) targeted by V2 antibodies in RV144 [6, 7] is underlined. Amino acids in this hotspot that differ from TH023.6 and A244 are shown in boldface type. Lysine (K) at position 169 and valine (V) at position 172 are indicated by a solid diamond. The sequence for GNE8 used in VAX003 is shown as a reference but was not made as a gp70-V1V2 to assess complement activation.

V1V2-specific complement-activating antibodies were measured as a function of C3d deposition on a panel of gp70-V1V2-coated beads. C3d was chosen for detection because it has been used previously to quantify complement activation [50], and because it is a dominant C3 cleavage fragment on complement-opsonized HIV-1 virions as determined in CD21-dependent binding experiments [42, 43]. Carboxylated beads were coated with either gp70 as a control for nonspecific activity, or with gp70 scaffolds containing the V1V2 domains of the gp120 vaccine immunogens used in RV144 (92TH093, A244 and MN) (Fig 1). A related heterologous V1V2 of the clade C strain, Ce1086, was included because of sequence similarity to 92TH023 and A244 (Fig 1) and because plasma IgG to this V1V2 correlated with a lower risk of HIV-1 infection in RV144 [3]. Notably, 92TH023 and A244 share an identical sequence in V2 that has been shown to be the target of RV144 V1V2 antibodies [6, 7], whereas Ce1086 differs by only two amino acids in this region (Fig 1). Assays were performed in the presence of NHS as a source of complement. In some cases a parallel set of assays was performed in the presence of C3-deficient human serum to confirm a requirement for complement. Optimization and equivalency of bead coating was performed using the rat anti-gp70 mAb K10-A11 and a biotin-conjugated anti-rat antibody (Southern Biotech, Cat. No. 6420–08). C3d deposition was quantified with biotinylated C3d monoclonal antibody and PE-Streptavidin.

### Complement activation by V1V2 mAbs from RV144

Four V1V2 mAbs (CH58, CH59, HG107 and HG118) from vaccine recipients in RV144 were evaluated for complement activation: CH58, CH59 and HG107 neutralize the highly sensitive tier 1A virus, 92TH023.6, but possess no neutralizing activity against tier 2 circulating strains [15]; HG118 is non-neutralizing (unpublished). Complement activation was assessed using gp70-V1V2 scaffolds containing V1V2 of TH023, A244, MN and Ce1086 and assayed in the presence of NHS as a source of complement. Each assay included beads coated with gp70 as a negative control. A separate set of assays was performed in parallel in C3-deficient human serum. As shown in Table 1, no activity was detected with gp70 assayed in the presence of NHS, or with gp70-V1V2 scaffolds assayed in C3-deficient serum, indicating that any positive activity against the scaffolds in the presence of NHS would be dependent on V1V2 and complement. In the presence of NHS, all four mAbs activated complement when beads were coated with gp70-V1V2 92TH023 (V1V2 of the vaccine strain used in vCP1521). Variable activity was seen with beads coated with gp70-V1V2 A244 (V1V2 of gp120 used as a protein boost in RV144). Weak to moderate activity was detected with gp70-V1V2 Ce1086. No activity was detected with gp70-V1V2 MN (another gp120 used for boosting in RV144) and the gp70 control. These results demonstrate that vaccine-elicited V1V2 IgG is capable of mediating complement activation, where the rank-order of positive activity was 92TH023>A244>>Ce1086. The results generally agreed with the ability of the antibodies to bind the scaffolds (Table 1), where greatest binding was seen against 92TH023 and A244, followed by Ce1086, and no binding was detected against MN. Interestingly, high binding to gp70-V1V2 A244 did not always predict strong complement activation.

**Table 1:** V1V2-specific monoclonal antibodies from RV144 activate complement.

		Mean fluorescence intensity				
		gp70-V1V2	gp70-V1V2	gp70-V1V2	gp70-V1V2	
mAb^1^	Assay	92TH023	A244	MN	Ce1086	gp70
CH58	Complement^2^	3087/9	153/12	15/12	153/11	22/17
	Binding^3^	44988	48979	70	9845	164
CH59	Complement	4434/9	719/12	15/12	265/11	22/16
	Binding	46417	50209	63	14884	211
HG107	Complement	5652/9	1521/12	16/11	532/11	24/16
	Binding	47000	50570	61	14184	158
HG118	Complement	2741/9	17/12	15/11	15/10	22/16
	Binding	19663	27356	45	195	51

^1^Monoclonal antibodies (mAb) were assayed at 17 μg/ml.

^2^Mean fluorescence intensity (MFI) of C3d detection in the presence of normal human serum (NHS) as a source of complement/MFI in the presence of C3-deficient human serum.

^3^MFI of antibody detection in the absence of human serum.

### Complement activating V1V2 antibodies in RV144

**Fig 2:**
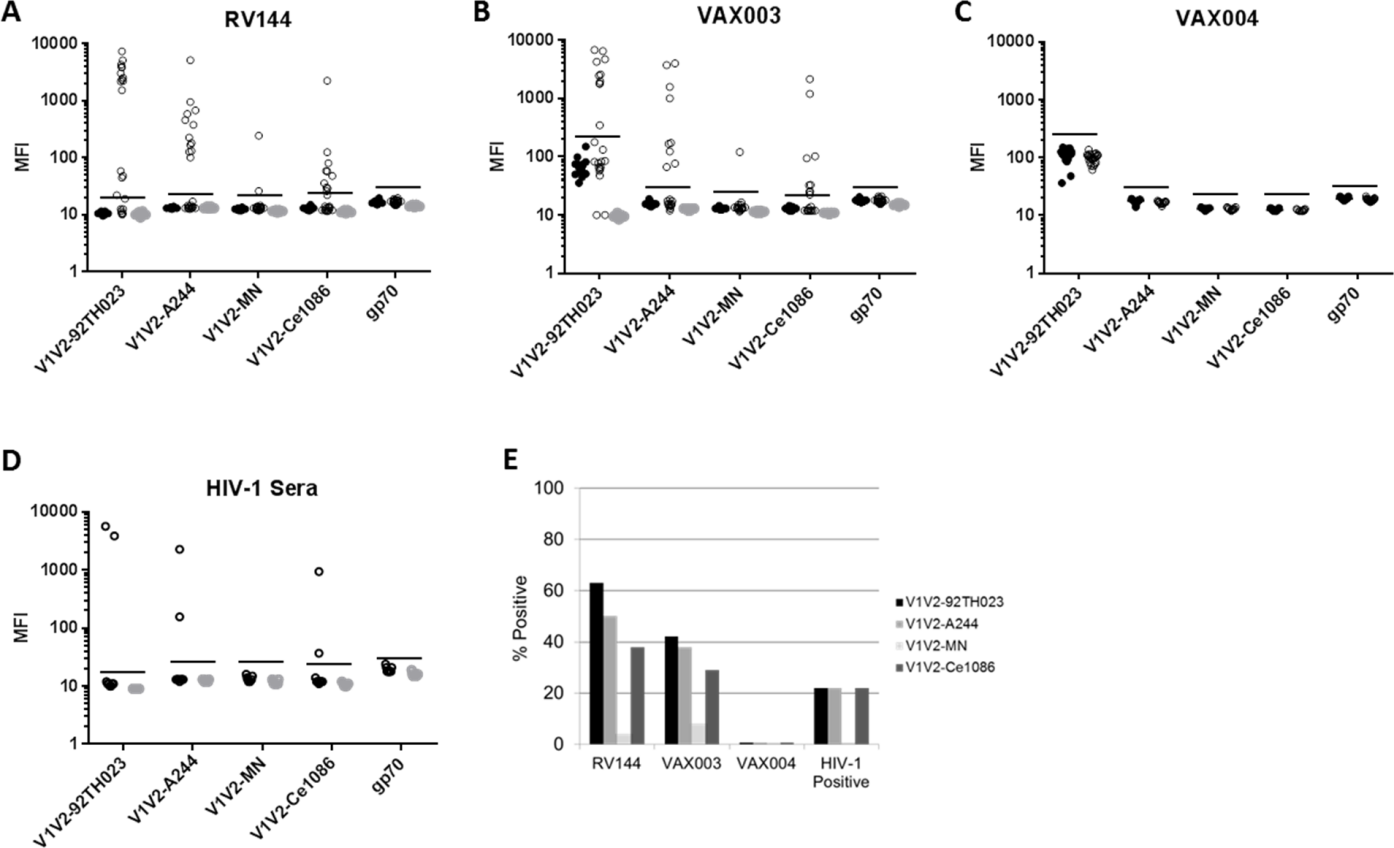
V1V2-specific complement activating antibodies in vaccine recipients and HIV-1-infected individuals. Samples obtained post-immunization or post-infection were assayed with gp70 and gp70-V1V2 scaffolds in the presence of either NHS as a source of complement (black open circles) or C3-deficient human serum (gray circles). Pre-immunization samples from vaccine recipients were assayed in the presence of normal human serum (black solid circles). Magnitude of complement activation is expressed as mean fluorescence intensity (MFI) of C3d detection for RV144 (**A**), VAX003 (**B**), VAX004 (**C**) and HIV-1-infected individuals (**D**). Note that post-immunization samples in VAX004 were not assayed in the presence of C3-deficient human serum. **E.** Percent of positive reactions in RV144, VAX003, VAX004 (n = 24 each) and CRF01_AE HIV-1-infected individuals (n = 9). Values >2x the highest negative control value were considered positive (values above the horizontal lines). Pre-immune sera from vaccine recipients assayed in the presence of NHS served as negative controls for RV144, VAX003 and VAX004. Assays in the presence of C3-deficient human serum served as negative controls for HIV-1-infected individuals.

**Fig 3:**
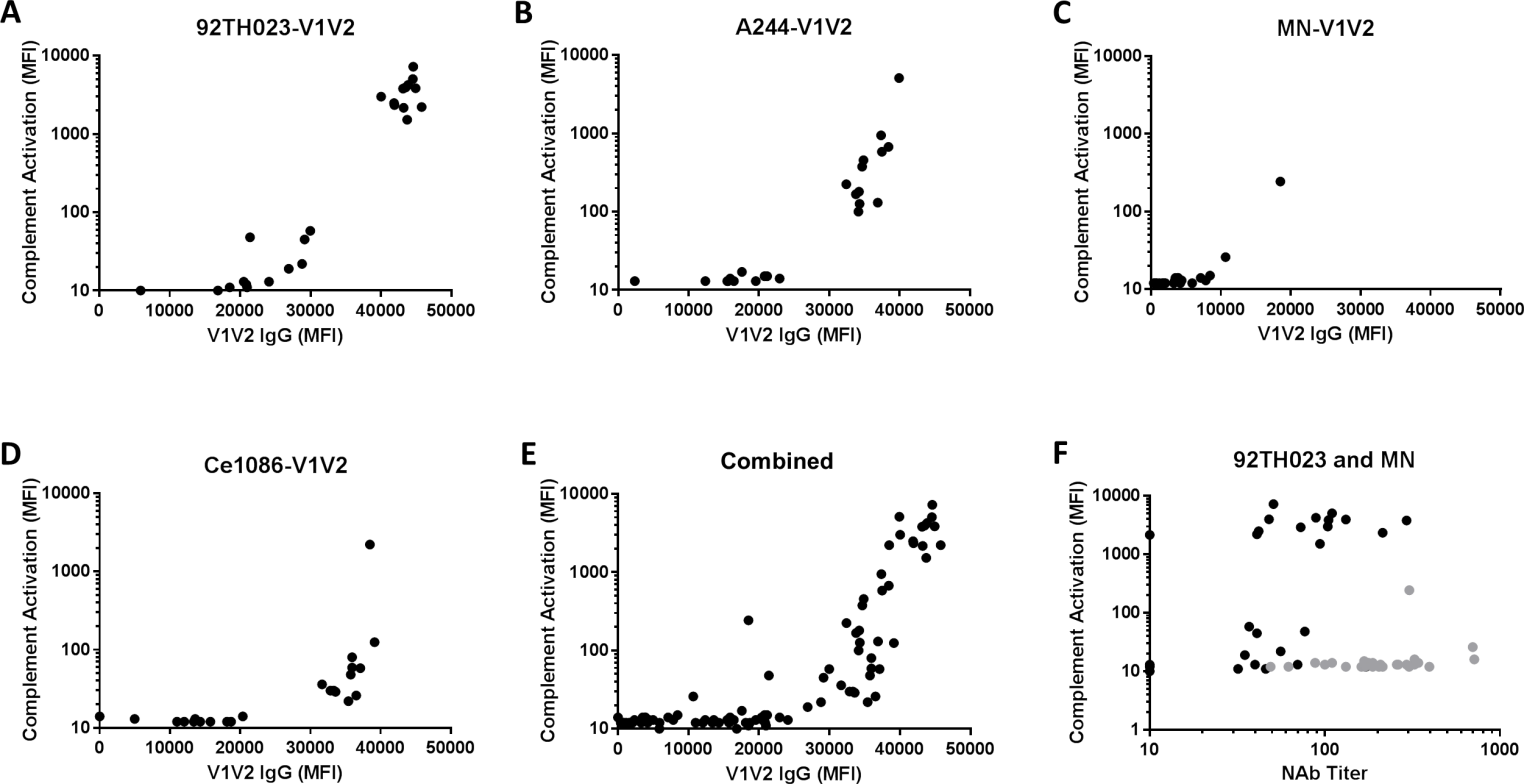
Complement activation requires a critical threshold level of V1V2-specific IgG. MFI values for complement activation by RV144 post-immune plasmas (visit 8) assayed in the presence of NHS were adjusted for non-specific activity by subtracting the MFI of corresponding pre-immune samples (visit 1) assayed in NHS (both MFI values in Fig 2A). The adjusted MFI values are plotted against the corresponding MFI values for V1V2-specific IgG in post-immune samples (after subtraction of non-specific IgG binding activity in pre-immune samples). Shown are results for individual antigens (A-D) and aggregate results (E) pooling over the four antigens for gp70-V1V2 of 92TH023, A244, MN and Ce1086. (F) Adjusted MFI values are plotted against corresponding neutralization titers against 92TH023 (black) and MN (grey).

We next assessed whether V1V2 antibodies in plasma samples from vaccine recipients in RV144 were capable of activating complement. Corresponding pre-immune (visit 1) and post fourth immunization (visit 8) plasmas from 24 vaccine recipients were analyzed. As shown in Fig 2A, variable and sometimes strong activity was detected with post-immunization samples (visit 8) and only against gp70-V1V2 scaffolds assayed in the presence of NHS. Like the V1V2 mAbs from RV144, activity was strongest against V1V2 of 92TH023, followed by A244 and Ce1086. Only one sample tested positive against MN, where this sample had the highest magnitude of MN-V1V2 binding IgG (Fig 3C). Pre-immune (visit 1) plasmas were consistently negative under all conditions. These results confirm that vaccination in RV144 elicited V1V2-specific complement activating antibodies.

Notably, a critical threshold of V1V2 IgG binding was required for complement activation. As seen in Fig 3, activation was weak or undetectable when the V1V2 IgG binding MFI was <30000, whereas all samples that had MFI >40000 were strongly positive. When the aggregate results in Fig 3E were analyzed, V1V2 IgG binding and complement activation were significantly associated with one another (Spearman r = 0.83). No significant association was seen between complement activation and neutralization titers against Env-pseudotyped viruses TH023.6 and MN.3 (Fig 3F), which are the two Tier 1A viruses most commonly neutralized by RV144 plasmas [10].

### Complement activating V1V2 antibodies in VAX003, VAX004 and HIV-1 infected individuals

A related clinical trial, VAX003, tested the efficacy of AIDSVAX B/E gp120 in the absence of vCP1521 priming and showed no evidence of any protection [45]. For comparison to RV144, pre-immune (visit 2) and post fourth immunization (visit 9) sera from 24 vaccine recipients in VAX003 were assayed for V1V2-specific complement activation. The results (Fig 2B) resemble those in RV144 in rank-order (refer to Fig 2A) except that the frequency of positive responses was consistently lower in VAX003 (Fig 2E). An unusually high frequency of moderate non-specific activity was seen in pre-immune samples from VAX003 when assayed against gp70-V1V2 92TH023. This non-specific activity was not seen in the presence of C3-deficient human serum, or when gp70 was assayed in the presence of NHS, indicating that it was both complement-dependent and V1V2-specific. Nonetheless, clear positive responses of much greater magnitude were observed in Vax003. Antibody-independent complement activation by HIV-1 has been described and shown to be influenced in part by terminal sialylation of N-linked glycans, where the presence of sialic acid blocks complement activation [43]. The V1V2 scaffolds used here contain several potential N-linked glycosylation sites, and each scaffold is different in the number and location of these sites (Fig 1). A unique feature of the glycans on 92TH023-V1V2 might have given rise to nonspecific activity. Additionally, we note that the samples from Vax003 and Vax004 had been stored for approximately 6 years longer than the samples from RV144, making it possible that longer-term storage contributed in part to a moderate level of nonspecific activity.

Another related clinical trial, VAX004, tested the efficacy of AIDSVAX B/B gp120 and, similar to Vax003, showed significant protection [46]. The gp120 in VAX004 differed from RV144 and VAX003 by replacing A244gp120 (CRF01_AE) with GNE8gp120 (clade B). We assayed pre-immune (visit 2) and post fourth immunization (visit 9) sera from 24 vaccine recipients in VAX004 and detected no V1V2-specific complement activating antibodies (Fig 2C and 2E). We did not use vaccine-matched GNE8-V1V2, which may partially explain the negative results. The negative results with MN-V1V2 confirm the poor immunogenicity of the V1V2 loop of MNgp120. Overall these results agree with the paucity of V2 binding antibodies detected in VAX004 [4]. Notably, V2 antibodies are also relatively uncommon in HIV-1-infected individuals [4]. In agreement with this, V1V2-specific complement activation was seen in only 2/9 CRF01_AE HIV-1 serum samples (Fig 2D and 2E).

### V1V2-specific complement activation by RV144 case-control plasmas

**Fig 4:**
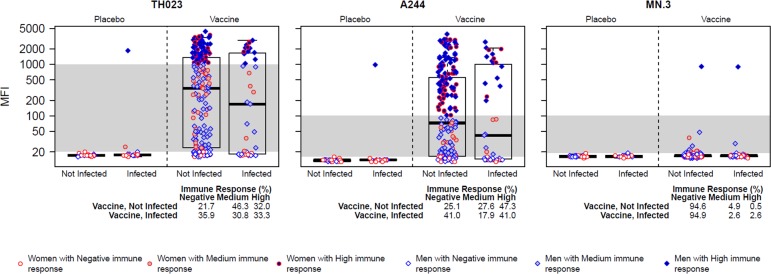
RV144 case-control assays for V1V2-specific complement activating antibodies. Plasma obtained 2 weeks post final boosting (week 26) from 41 vaccine recipients (cases) who acquired HIV-1 infection after week 26, and from an additional 205 vaccine recipients (controls) who had not acquired infection by the end of the trial (month 42) were assayed for complement activation against V1V2 of the three vaccine strains (92TH023, A244 and MN). Week 26 plasma from 20 placebo recipients who acquired HIV-1 infection after week 26, and 20 placebo recipients who remained uninfected at the end of the trial were included as negative controls. Readouts are shown as box plots of reactivity with each of the 3 V1V2-scaffold antigens with plasma from the 286 case-control specimens. Box plots show the 25th percentile (lower edge of the box), 50th percentile (horizontal line in the box), and 75th percentile (upper edge of the box). Participants are stratified according to HIV-1 infection status and treatment assignment. Gender and immune response categories are indicated by the color and shape of the points. Negative, Medium, and High are defined are defined in the Methods section and are divided by the gray shaded horizontal bands.

**Fig 5:**
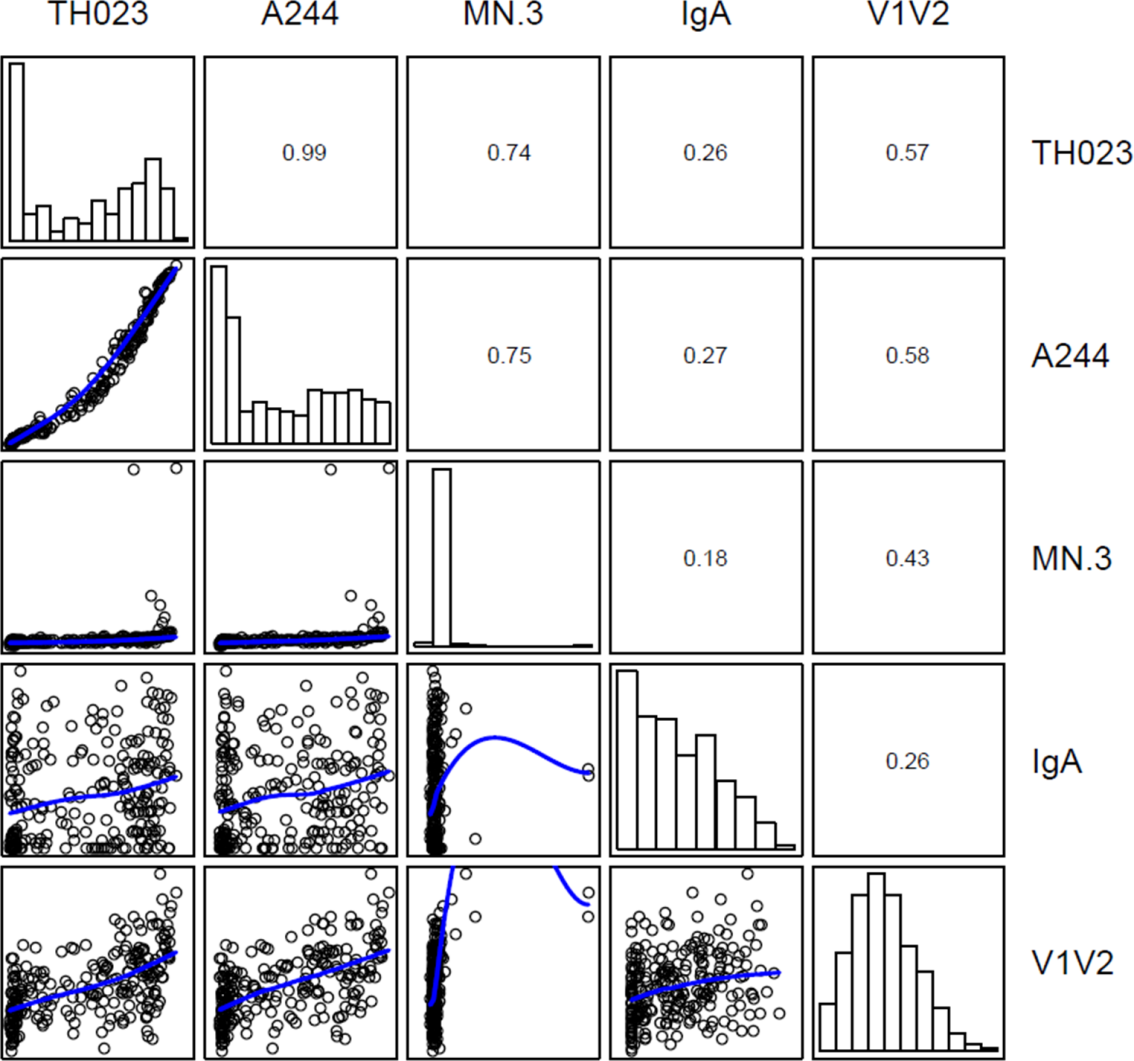
Correlation between RV144 case-control readouts for three V1V2-specific complement activating antibody specificities (92TH023, A244, MN) and primary IgA and IgG V1V2 readouts. The log-transformed MFI or OD values are displayed in pairwise scatter plots below the diagonal or as a histogram on the diagonal. For each pair of readouts the Spearman correlation is displayed above the diagonal. Scatter plots include a blue loess smooth line. The primary IgA and IgG V1V2 primary variables were studied in the original case-control analysis [2].

Our finding that RV144 V1V2 antibodies activate complement raised the question of whether this activity would correlate with a reduced risk of HIV-1 infection. To address this question, case-control plasma samples from RV144 were assayed in NHS with gp70 alone and gp70 scaffolds containing V1V2 derived from the three vaccine strains. All assays were performed blinded with respect to infection status and immunization group. As seen in Fig 3 (also S1 Table), plasmas from vaccine recipients exhibited a wide range of activity against 92TH023-V1V2 and A244-V1V2, while little or no activity was seen against MN-V1V2; results with gp70 were all negative. As expected, plasma samples from placebo recipients were mostly negative with all antigens. Although the median activity against V1V2 of 92TH023 and A244 trended higher in non-infected vaccine recipients than in infected vaccine recipients (Fig 4), the quantitative activity levels (comparison of all MFI values between groups) did not statistically significantly associate with HIV-1 infection (Table 2). However, we did find a significant inverse correlation with HIV-1 infection risk when analyzing the RV144 vaccine matched antigens (TH023-V1V2 and A244-V1V2) as dichotomous readouts. Since these two antigens had the highest response rates when assayed against serum from RV144 vaccine recipients as compared to VAX003, VAX004 and HIV-1 positive sera, we tested positive versus negative response as a correlate of risk. In the model adjusting for both the primary IgA and primary IgG V1V2 readouts, vaccine recipients with positive 92TH023-V1V2-specific complement activating antibodies had a significantly lower infection risk than vaccine recipients with negative response (OR = 0.42, P-value = 0.048, Table 3), and a similar marginally significant result was found for A244-V1V2 (OR = 0.49, P-value = 0.085, Table 3). To help interpret these results, the fact that they are similar for 92TH023-V1V2 and A244-V1V2 is explained by the very high inter-correlation of the readouts to these two antigens (Fig 5, Spearman r = 0.99). In addition, the complement 92TH023-V1V2-specific and A244-V1V2-specific activating antibodies were moderately correlated with the primary IgG V1V2 readout to scaffolded gp70-V1V2 case A2 (r = 0.57 and r– 0.58, respectively) and weakly correlated with the primary IgA variable (r = 0.26 and r = 0.27, respectively), showing that the complement activating antibodies can detect independent activity; the correlates results of Table 3 indicate that the complement antibodies are independent correlates after accounting for the two primary variables.

**Table 2:** Results testing the correlation of quantitative complement activity readouts with HIV-1 infection.

V1V2 Complement Variable	Model^1^	OR (95% CI) per SD	P-Value
TH023	Univariate	0.84 (0.59–1.19)	0.31
	IgA	0.77 (0.54–1.11)	0.16
	IgA+V1V2	0.98 (0.64–1.48)	0.91
A244	Univariate	0.90 (0.64–1.28)	0.57
	IgA	0.83 (0.58–1.20)	0.32
	IgA+V1V2	1.10 (0.72–1.69)	0.66
MN.3 Univariate	Univariate	1.11 (0.84–1.45)	0.46
	IgA	1.08 (0.82–1.43)	0.57
	IgA+V1V2	1.30 (0.95–1.78)	0.096

^1^All models adjust for baseline behavioral risk score and gender, and report the odds ratio (OR) of HIV-1 infection per SD increase in the V1V2 complement variable. The “IgA” and “IgA+V1V2” models adjust for the primary IgA and V1V2 variables studied in the original case-control analysis (2) whereas the “Univariate” models do not adjust for these immune response variables.

**Table 3:** Results testing the correlation of complement response call readouts with HIV-1 infection for all models and all variables analyzed.

V1V2 Complement Variable	Model^1^	OR (95% CI)	P Value
TH023	Univariate	0.42 (0.20, 0.87)	0.02
TH023	IgA	0.31 (0.14, 0.70)	0.005
TH023	IgA+V2	0.42 (0.18, 0.99)	0.048
A244	Univariate	0.41 (0.20, 0.85)	0.016
A244	IgA	0.36 (0.17, 0.77)	0.008
A244	IgA+V2	0.49 (0.21, 1.10)	0.085

^1^All models adjust for baseline behavioral risk score and gender, and report the odds ratio (OR) of HIV-1 infection comparing Positive versus Negative response to the V1V2 complement variable. The “IgA” and “IgA+V1V2” models adjust for the primary IgA and V1V2 variables studied in the original case-control analysis (2) whereas the “Univariate” models do not adjust for these immune response variables.

In order to understand whether or not the strongly positive complemenat activation responses observed in the assay correlated with infection risk, we further split positive responses into “Medium” and “High” response based on the dichotomy observed while developing the assay. Interestingly, the strongest inverse correlation with risk was seen in the group of positive but not strongly positive responders (TH023-V1V2: OR = 0.33 for Medium vs. Negative, P-value = 0.019; A244-V1V2: OR = 0.36 for Medium vs. Negative, P-value = = 0.049; Table 4) while strong positive responses were not significantly correlated with risk (TH023-V1V2: OR = 0.73 High vs. Negative, P-value = 0.56; A244-V1V2: OR = 0.64 High vs. Negative, P-value = 0.35; Table 4).

**Table 4:** Results testing the correlation of categorical complement readouts with HIV-1 infection for all models and all variables analyzed.

V1V2 Complement Variable	Model^1^	Contrast	OR (95% CI)	P Value	Global P Value^2^
TH023	Univariate	Med vs. Neg	0.33 (0.14, 0.77)	0.01	0.038
		High vs. Neg	0.55 (0.24, 1.30)	0.17	
TH023	IgA	Med vs. Neg	0.26 (0.10, 0.64)	0.003	0.012
		High vs. Neg	0.40 (0.16, 1.00)	0.051	
TH023	IgA+V2	Med vs. Neg	0.33 (0.13, 0.84)	0.019	0.043
		High vs. Neg	0.73 (0.25, 2.11)	0.56	
A244	Univariate	Med vs. Neg	0.32 (0.12, 0.85)	0.022	0.044
		High vs. Neg	0.47 (0.21, 1.02)	0.057	
A244	IgA	Med vs. Neg	0.31 (0.11, 0.84)	0.022	0.027
		High vs. Neg	0.39 (0.17, 0.89)	0.025	
A244	IgA+V2	Med vs. Neg	0.36 (0.13, 1.00)	0.049	0.14
		High vs. Neg	0.64 (0.25, 1.63)	0.35	

^1^All models adjust for baseline behavioral risk score and gender, and report the odds ratio (OR) of HIV-1 infection comparing High (or Medium) versus Negative response to the V1V2 complement variable. The “IgA” and “IgA+V1V2” models adjust for the primary IgA and V1V2 variables studied in the original case-control analysis (2) whereas the “Univariate” models do not adjust for these immune response variables. MN-V1V2 data were not analyzed because there was no clear distinction between medium and high responses.

^2^Generalized Wald test of the null hypothesis that HIV-1 risk is the same across the Negative, Medium, and High subgroups versus the alternative hypothesis that there is some difference.

We also asked whether the HIV-1 risk correlation with complement activation differs across the levels of primary IgG binding to gp70-V1V2 case A2. We carried out this analysis, for two subgroups of vaccine recipients defined by the primary IgG gp70-V1V2 Case A2 readout being above (High) or below (Low) the median readout. Because of reduced sample size for these subgroup analyses, only primary IgG was adjusted for in these models (not baseline behavioral risk score and gender as in Table 2). The results are reported in Table 5 and show that the complement readout is not associated with HIV-1 infection within either the Low or High primary gp70-V1V2 Case A2 subgroups (all p-values ≥ 0.15). Our interpretation of this result is that after stratifying by the gp70-V1V2 Case A2 readout, there is limited variability in the complement readout, which reduces statistical power to detect an association between complement and HIV-1 infection.

**Fig 6:**
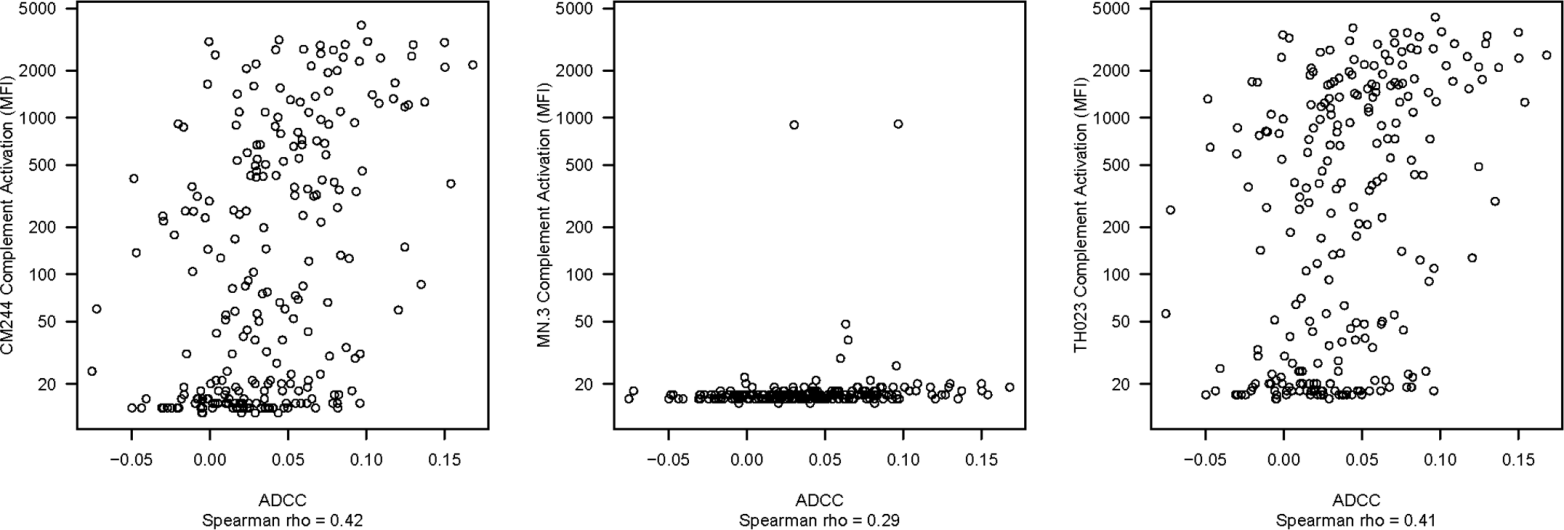
Association of complement activation with the primary ADCC variable in RV144. Shown are associations between complement activation and ADCC activity against CM2544, MN.3 and TH023.

Finally, we asked whether complement activation was associated with the ADCC activity of the case-control samples. As shown in Fig 6, only weak positive correlations ranging from Spearman rank r = 0.29 to 0.42 were seen.

**Table 5:** Complement readout logistic regression model correlates of risk assessment within vaccine recipient subgroups with Low and High primary gp70-V1V2 Case A2 binding antibodies^1^.

Primary gp70-V1V2 Case A2 Subgroup	Antigen	Complement Predictor Type	Est. Odds Ratio of Complement Readout	Lower limit 95% CI	Upper limit 95% CI	2-sided p-value
Low^2^ primary V1V2	TH023	Quantitative^3^	0.83	0.52	1.31	0.42
High primary V1V2	TH023	Quantitative	1.42	0.75	2.70	0.29
Low primary V1V2	CM244	Quantitative	0.84	0.53	1.35	0.48
High primary V1V2	CM244	Quantitative	1.58	0.85	2.95	0.15
Low primary V1V2	TH023	Binary^4^	0.66	0.28	1.57	0.34
High primary V1V2	TH023	Binary	0.43	0.080	2.29	0.32
Low primary V1V2	CM244	Binary	0.62	0.26	1.47	0.27
High primary V1V2	CM244	Binary	0.49	0.094	2.60	0.40

^1^The logistic regression model adjusts for the primary IgA variable studied in Haynes et al. (2012).

^2^The two subgroups of vaccine recipients are defined by the primary gp70-V1V2 case A2 readout being above or below the median value.

^3^The quantitative complement readout variable is the same as used in the main article, with mean zero and standard deviation one within each of the Low and High primary V1V2 strata.

^4^The binary complement readout is defined by the same complement positive response call used in the main article, where the odds ratio is for positive response versus negative response.

## Discussion

We sought to determine whether the V1V2-specific IgG responses that correlated with a decreased risk of HIV-1 infection in RV144 were associated with a possible complement-mediated mechanism of protection. HIV-1 resists terminal complement pathway activation and MAC formation; however, early pathway activation and opsonization by activated C3 fragments could promote interactions with complement receptors on follicular dendritic cells and red blood cells to facilitate antibody responses in germinal centers [51, 52] and HIV-1 clearance through the mononuclear phagocytic system [40, 41]. As indicated by C3d deposition on V1V2 antigen-coated beads, plasma samples obtained two-week post final vaccination in RV144 exhibited variable levels of complement activation. The magnitude of antibody-mediated complement activation correlated with V1V2 IgG and was most pronounced against V1V2 of the vaccine strain in vCP1521 (92TH023) and to a lesser degree V1V2 of A244gp120 used in the protein boost. The V1V2 of these two CRF01_AE strains are closely related, though not identical, and share a common sequence in the RV144 V2-reactive hotspot region (Fig 1). Notably, they differ by four glycans and a number of non-sequon amino acids over the entire length of the scaffold, including a six amino acid insertion in A244. Minor differences in reactivity against these two V1V2 scaffolds may be due to differences in the balance of polyclonal specificities, or in the structure and glycan shielding of the V2 hotspot [15]. The near absence of activity against V1V2 of the clade B MNgp120 that was also used in the protein boost is likely explained by low IgG levels against this more divergent V1V2. Moderate activity was seen against the non-vaccine-matched Ce1086-V1V2, which shares partial sequence homology to 92TH023 and A244, especially in the RV144 V2-reactive hotspot region (Fig 1).

Complement activation by V1V2-specific antibodies was stronger and detected more frequently in RV144 than in two related trials, VAX003 and VAX004, which did not include vector priming and where no significant protection was observed. It was also more common to detect V1V2-specific complement activation in RV144 than in individuals who were chronically infected with CRF01_AE HIV-1. The superior complement activation seen in RV144 led us to test whether the activity correlated with a lower risk of HIV-1 infection. Gp70 scaffolds containing V1V2 of the three gp120 vaccine strains (92TH023, A244 and MN) were used to assay case-control plasmas. The results showed that vaccine recipients with positive complement activation to 92TH023-V1V2 and A244-V1V2 had a significantly lower HIV-1 infection risk than vaccine recipients without complement activation. This correlation is perhaps expected given the positive correlation between complement activation and V1V2-specific IgG (Fig 3), and the fact that previous results showed that a higher magnitude of IgG binding to one of the antigen used to measure complement activation, 92TH023-V1V2, correlated with a lower HIV-1 infection risk [3]. Interestingly, the inverse correlation of antibody-mediated complement activation seen with 92TH023-V1V2 and A244-V1V2 with risk persisted even after adjusting for IgG gp70-V1V2 Case A2 responses. Therefore for vaccine recipients with the same V1V2 Case A2 responses, those with complement activation had lower risk of HIV-1 than those without complement activation. We do not understand why the strongest inverse correlation with risk was seen in the group of complement activation positive but not strongly positive responders. Complement can mediate a variety of biologic functions, and it is possible that the balance of these functions is dependent on the level of activation.

Overall these results suggest that a certain level of antibody-dependent complement activity may have contributed in part to a reduced risk of HIV-1 infection in RV144. Additional studies are needed to verify this role of complement and to delineate the mechanism. The mechanism might be part of a balanced polyfunctional antibody response that also includes FcR-mediated effector functions, which altogether may be needed for protection.

## Supporting information

S1 Table Complement activation by case-control plasmas from RV144. (XLSX)
